# Determinants of Home Delivery among Mothers in Abobo District, Gambella Region, Ethiopia: A Case Control Study

**DOI:** 10.1155/2020/8856576

**Published:** 2020-12-09

**Authors:** Asmelash Abera Mitiku, Abraham Lomboro Dimore, Solomon Berhanu Mogas

**Affiliations:** ^1^Gambella Regional Health Bureau, Gambella Region, Ethiopia; ^2^Department of Epidemiology, Jimma University, Jimma, Oromia, Ethiopia

## Abstract

**Introduction:**

Home delivery is one of the major reasons for high maternal mortality ratio in sub-Saharan Africa. Sub-Saharan Africa and South Asia together contribute over 85% of maternal deaths, of which, only half of deliveries are institutional. However, data are scarce on the availability of information with regard to the determinant factors for this high prevalence of home delivery in the study area.

**Objective:**

This study is aimed at determining factors associated with home delivery, among mothers in Abobo Woreda, Gambella region, Southwest Ethiopia, 2019.

**Methods:**

A case control study conducted from 12 March 2019 up to 2 April 2019 on 88 cases and 176 controls. Cases include mothers who gave birth at home and those mothers who gave birth at health facility in the last one year preceding the study included as controls. Data entry was made using Epi-Data version 3.1, and analysis was made using SPSS version 20. A binary logistic regression analysis was conducted to assess candidate variables and subsequently a multivariable regression to determine the statistical associations. Adjusted odds ratio (AOR) with 95% confidence interval (CI) was calculated to determine strength of association, and *p* value <0.05 was used to establish significant associations.

**Results:**

No formal education (AOR: 5.07; 95% CI: 2.18-11.50), poor knowledge on obstetric complications (AOR: 3.83; 95% CI: 1.98-7.40), negative attitude towards delivery service (AOR: 3.25; 95% CI: 1.70-6.19), poor household wealth index (AOR: 4.55; 95% CI: 2.01-10.31), and no antenatal care visit (AOR: 3.29; 95% CI: 1.63-6.63) were found to be significantly associated with home delivery.

**Conclusions:**

The findings do support that no formal education, poor knowledge on obstetric complications, negative attitude towards delivery service, poor household wealth index, and no antenatal care visit showed a significant association with home delivery.

## 1. Introduction

Even though, most pregnancies and births are uneventful, too many women still suffer and die from serious health issues during pregnancy and childbirths [1]. Maternal mortality (MM) is not merely due to particular pathologies but has strong association with women's social determinants of health [2]. The target of Sustainable Development Goal (SDG 3.1) is to reduce global maternal mortality ratio (MMR) to less than 70 deaths per 100,000 live births with annual rate of 7.3% reduction and with a maximum target of no more than 140 deaths per 100,000 live births for any country [1].

Globally, due to pregnancy related causes, an estimated 303,000 maternal deaths occurred every year. From the total deaths, 99% were from developing countries and 66% in sub-Saharan Africa alone [3, 4]. According to the Ethiopian Demographic and Health Survey (EDHS) 2016 report, the MMR in Ethiopia was 412/100,000 live births [5].

It has become increasingly clear that MMR in sub-Saharan Africa is highly attributed to home delivery, and most births take place at home [2]. Globally, about two-thirds of births take place in the HF [6]. However, in developing countries, home delivery is widely practiced. Mothers deliver in unhygienic environment, without skilled birth attendant and lifesaving medications [7]. Sub-Saharan Africa and South Asia together contribute over 85% of maternal deaths, and of which only half of deliveries are institutional [6]. Previous studies across the sub-Saharan Africa report a significant proportion of mothers still deliver at home [8–10]. Furthermore, a cross-sectional study conducted in Abobo District showed that significant number of women (91.5%) had given birth at home [11]. There is enthusiasm by the government to reduce the high prevalence of home delivery in Ethiopia. However, data are scarce on the availability of information with regard to the determinant factors for this high prevalence of home delivery. Therefore, given the scarcity of studies conducted to examine predictors as to why women give birth at home, the current study adds potential determinant factors (enabling, predisposing, and need based) for home delivery in rural settings to current literatures. In addition to this, the finding from the current study provides evidence for decision makers, stakeholders, and health professionals aimed to reduce MMR by decreasing home delivery specifically in the study area and generally to areas with similar rural settings. Therefore, the study is aimed at identifying determinants of home delivery in the study area.

## 2. Materials and Methods

### 2.1. Study Setting, Design, and Population

The study was conducted at Abobo District, Anguwa Zone, Gambella region state, Southwest Ethiopia. The district has 19 kebeles (the smallest administrative unit in Ethiopia) with a total population of 31,209 (15,292 males and 15,917 females). Among the total population, 8,146 women were in the reproductive age group. There are four health centers found in the district and providing child delivery services for the population.

A community-based case control study was conducted. The study population includes mothers who gave birth in the last one year preceding the current study. Cases were permanent resident mothers who gave birth in the last one year outside of health institutions irrespective of the delivery attendant. Controls were mothers who gave birth in the last one year in health institutions (health center in this case) irrespective of the delivery attendant. The current study was conducted from March 12 to April 2, 2019.

### 2.2. Sampling

Sample size was calculated using the Epi Info version 7 software program by considering 80% power, 95% confidence level, 10% nonresponse rate, a1 : 2 case to control ratio, and taking exposure factors from different studies [12–14]. The sample size was 264 (88 cases and 176 controls).

Stratified random sampling technique was used to select the study participants. All the 19 kebeles (two urban and 17 rural) found in Abobo Woreda were taken. Then, sampling frame was prepared for cases and controls separately for each kebele with their corresponding household identification numbers by making house-to-house survey. Sample size was allocated by proportional to the size allocation (PPS) of each kebele. Finally, 88 cases and 176 controls were selected by simple random sampling technique within each strata using computer-generated random number by Excel sheet.

### 2.3. Data Collection

Face to face interview technique was used to collect data by making house to house visit. The questionnaire was adapted from different literatures and Ethiopian Demographic and Health Survey (EDHS) [5, 12, 14]. Data were collected by using seven trained diploma nurses.

### 2.4. Measurement

*Knowledge on danger signs of pregnancy*: those who mentioned at least four danger signs of pregnancy were classified as having good knowledge.

*Knowledge on obstetric complications of labor or delivery*: those who mentioned at least three obstetric complications were classified as having good knowledge.

*Attitude towards delivery services*: those who scored greater than or equal to the mean were labeled as having positive attitude [14].

### 2.5. Operational Definitions

*Predisposing factors*: this implies the proclivity to utilize health care services. An individual is more or less likely to use health services based on demographics, position within the social structures, and beliefs of health services benefits.

*Enabling factors*: this implies resources found within the family and the community.

*Need-based factors*: this includes the perception of need for health services, whether individual, social, or clinically evaluated perceptions of need.

*Household wealth index*: this includes household ownership of selected assets, such as televisions, bicycles, housing constructions, type of water access, and sanitation facilities.

### 2.6. Quality Control

Survey was conducted to prepare sampling frame for cases and controls. Diploma nurses who have been working in the study area were recruited to collect data. To increase the quality of data, training was given to data collectors on the objective and data collection tools of the study.

### 2.7. Data Analysis

Data cleaning, coding, and entry were made using the Epi-Data version 3.1 software. Analysis was conducted using Statistical Package for Social Science (SPSS) version 20. To determine associations between dependent and independent variables, logistic regression analysis was used. Bivariate analysis was performed to select variables for multivariable analysis; *p* value <0.25 was used as a cutoff point. Multivariable logistic regression was used to determine significant association between the outcome and independent variable. Multicollinearity diagnostic was done by checking variance inflation factor (VIF), and no problems were identified. Backward stepwise logistic regression was used to determine independent predictors with *p* value <0.05 with their respective AOR and 95% CI. The model fitness was checked by Hosmer-Lemeshow goodness of fit test, and the model was declared as fit model since *p* value was greater than 0.05.

### 2.8. Ethical Clearance

Ethical clearance was obtained from Jimma University Institute of Health Ethical Review Committee. Permission letters were also obtained from regional health bureau and Abobo District health office. Confidentiality was assured by excluding their name during the period of data collection. The study purpose, procedure, duration, possible risks, and benefits of the study were clearly explained for the study participants. Before any kinds of data collection, informed verbal consent was obtained from each study participant.

## 3. Results

### 3.1. Sociodemographic Characteristics of Cases and Controls

In this study, a total of 88 cases and 176 controls were included with a response rate of 100% in both groups. Of total respondents, 47 (53.4%) cases and 58 (33.0%) controls were above age of 30 years. The median ± IQR (interquartile range) age of cases and controls were 30.00 ± 10 and 28.00 ± 6 years, respectively. About 60 (68.2%) cases and 121 (68.8%) controls were rural residents. Concerning educational status, 22 (25.0%) cases and 75 (42.6%) controls had secondary and above educational status. Regarding ethnicity, majority of the study participant 55 (62.5%) cases and 102 (58.0%) controls were Anyuak (Table 1).

### 3.2. Knowledge, Attitude, and Decision on the Place of Delivery

Thirty-four (38.6%) cases and ninety-eight (55.7%) controls had good knowledge about danger signs of pregnancy. Regarding knowledge about obstetric complications, 26 (29.5%) cases and 118 (67.0%) controls had good knowledge.

Regarding about attitude towards delivery services, six questions related to benefits of giving birth at HF, health professionals' skill, health services staffing, and availability of supplies were asked. Accordingly, 30 (34.1%) cases and 125 (71.0%) controls had positive attitude towards delivery services (Table 2).

## 4. Socioeconomic and Health Service-Related Factors

Concerning the household wealth index, 20 (22.7%) cases and 70 (39.8%) controls were in the rich wealth tertile (category).

Regarding to health service-related characteristics, majority of cases 87 (98.9%) and controls 173 (98.3%) mentioned that there was health facility in their residence (Table 3).

## 5. Need-Based Factors for Cases and Controls

Of total respondents, 40 (45.5%) cases and 142 (80.7%) controls had history of ANC visit for her last pregnancy. From those who had ANC visit, 14 (35.0%) cases and 103 (72.5%) controls had four and above number of visit. Regarding to previous obstetric outcome, 80 (90.9%) cases and 156 (88.6%) controls reported that they had not faced any obstetric problems during their last deliveries (Table 4).

### 5.1. Determinants of Home Delivery among Mothers

We found five variables significantly associated with home delivery: mothers who had no formal education (AOR: 5.07; 95% CI: 2.18-11.50), mothers who had poor knowledge on obstetric complication (AOR: 3.83; 95% CI: 1.98-7.40), mothers who had negative attitude towards delivery service (AOR: 3.25; 95% CI: 1.70-6.19), mothers who were in the poor wealth tertile (AOR: 4.55; 95% CI: 2.01-10.31), and mothers who had no ANC visit (AOR: 3.29; 95% CI: 1.63-6.63) (Table 5).

## 6. Discussion

This study, the first in the district, has identified factors that could predict home delivery among mothers in Abobo District of Gambella regional state. Our study has demonstrated results that are consistent with other studies in the literature. Accordingly, predisposing factors (educational status of mothers, knowledge about obstetric complications, and attitude of mothers towards delivery services), enabling factors (household wealth index), and need-based factors (ANC visit) were identified as predictors of home delivery.

Accordingly, those mothers who had no formal education had a significant association with home delivery than mothers who had secondary and above education. This finding is in line with other studies conducted in Bahirdar Ethiopia, South Tigray zone of Northern Ethiopia, Chandigarh of India, and Ghana [12, 13, 15, 16]. The observed difference could be level of awareness difference between the two groups. This highlights the need to educate mothers and the community as a crucial intervention step for substantial reduction of MMR.

Furthermore, the present study found that mothers' knowledge on obstetric complications had a significant association with home delivery. Accordingly, mothers who had poor knowledge on obstetric complications showed higher odds on home delivery than their counter parts. It is supported by different studies [14, 17–19]. As knowledge of mothers on obstetric complication increases, they prefer to deliver at health facilities. Teaching mothers and the community on obstetric complication increases the need for professional care and preference of place of delivery, as this is likely to be more helpful in assisting decision-making.

Mothers who had negative attitude towards delivery service disclosed higher odds on home delivery than mothers with positive attitude. This finding is consistent with a study conducted in Zala District, Southern Ethiopia [20]. Negative attitudes affect positive practices. Improving the attitude of mothers on facility delivery services can play a significant role on the preference of delivery services. The district health office needs to play its role on increasing awareness of mothers and the community towards delivery service.

In the present study, mothers in the poor household wealth category were more likely to give birth at home than mothers in the rich category. This finding is similar with different studies conducted in Kenya and Ethiopia [5, 21, 22]. Even if maternal health services are free, there are different costs related to transportation. As different studies showed, financial capability of families and costs related to transportation may not be afforded by mothers from poor households [23, 24]. To improve health facility delivery, free transportation mechanisms and access to health facilities need to be considered.

Further, the odd of home delivery was higher among mothers with no ANC visit. This finding is in agreement with studies conducted in Istanbul Turkey, South Tigray zone, and Tanqua-Abergele district, Tigray Region, Ethiopia [14, 15, 25]. ANC package includes health education and promotion works related to pregnancy and its complication and recommended practices. Since the study area was rural setting, the communities including mothers' access to information on maternal and related health issues are limited to health facilities. If mothers miss their opportunity for ANC services during their pregnancy period, their decision on selection of place of delivery will be affected. Due to this, the area health office needs to boost ANC service utilization.

## 7. Strengths and Limitations

This study is the first to examine determinant factors for home delivery. It used a community-based case control approach among mothers who gave birth during the past one year.

The study considers only mothers who gave birth in the last one year preceding the current study; the evidence might not reflect the scenario of periods prior to this. Since data collectors were health workers, social desirability bias was the other limitations of this study and it could have effect on the report of mothers. However, helping mothers to remember the events, adequate training given to data collectors, and their supervision could minimize the effects of recall bias and social desirability bias.

## 8. Conclusion

This study provides evidence on the determinants of home delivery in Abobo District. It confirms that predisposing factors (mothers' educational status, knowledge on obstetric complications, and attitude towards delivery services), enabling factors (household wealth index), and need-based factors (ANC visit) were positively and significantly associated with home delivery among mothers in Abobo District, Southwest Ethiopia. From the identified determinants, majority were predisposing factors. Hence, the regional and district health office and other stakeholders need to tackle home delivery by focusing on mother's educational status, knowledge on obstetric complications, and attitude towards delivery services.

## Figures and Tables

**Table 1:** Sociodemographic characteristics of cases and controls in Abobo District, Gambella region, Southwest Ethiopia, 2019.

Variables	Category	Cases (88) No. %	Controls (176) No. %
Age of mothers	15-24	19 (21.6)	47 (26.7)
	25-29	22 (25.0)	71 (40.3)
	≥30	47 (53.4)	58 (33.0)

Residence	Rural	60 (68.2)	121 (68.8)
	Urban	28 (31.8)	55 (31.3)

Educational status of mothers	No formal education	42 (47.7)	29 (16.5)
	Primary (1–8)	24 (27.3)	72 (40.9)
	Secondary and above	22 (25.0)	75 (42.6)

Educational status of husbands	No formal education	17 (19.3)	35 (19.9)
	Primary (1–8)	34 (38.6)	59 (33.5)
	Secondary and above	37 (42.0)	82 (46.6)

Occupational status of mothers	Housewives/farmer	65 (73.9)	126 (71.6)
	Students	9 (10.2)	22 (12.5)
	Merchants	5 (5.7)	13 (7.4)
	Government employee	9 (10.2)	15 (8.5)

Occupational status of husbands	Farmer	55 (62.5)	91 (51.7)
	Students	10 (11.4)	13 (7.4)
	Daily laborer	6 (6.8)	14 (8.0)
	Merchants	3 (3.4)	17 (9.7)
	Government employee	14 (15.9)	41 (23.3)

Family size	<5	36 (40.9)	79 (44.9)
	≥5	52 (59.1)	97 (55.1)

Parity	One birth	22 (25.0)	35 (19.9)
	Two birth	17 (19.3)	44 (25.0)
	Three birth	14 (15.9)	35 (19.9)
	Four birth	19 (21.6)	30 (17.0)
	Fifth and above birth	16 (18.2)	32 (18.2)

**Table 2:** Bivariate analysis of knowledge, attitude, and decision on place of delivery among cases and controls in Abobo Woreda, Gambella region, Southwest Ethiopia, 2019.

Variables	Category	Cases (88) No. %	Controls (176) No. %	COR (95% CI)	*p* value

Knowledge on danger sign of pregnancy	Poor	54 (61.4)	78 (44.3)	1.99 (1.18-3.36)	0.009^∗^
	Good	34(38.6)	98(55.7)	1	

Knowledge on obstetric complications	Poor	62 (70.5)	58 (33.0)	4.85 (2.78-8.46)	<0.001 ^∗^
	Good	26 (29.5)	118 (67.0)	1	

Attitude towards delivery services	Negative	58 (65.9)	51 (29.0)	4.74 (2.74-8.19)	<0.001^∗^
	Positive	30 (34.1)	125 (71.0)	1	

Decision on place of delivery	Self	26 (29.5)	49 (27.8)	0.97 (0.32-2.93)	0.961
	With husband	30 (34.1)	81 (46.0)	0.68 (0.23-1.99)	0.482
	Husband alone	26 (29.5)	35 (19.9)	1.36 (0.45-4.16)	0.588
	Others^∗^	6 (6.8)	11 (6.3)	1	

Note: ^∗^significant at *p* < 0.25; others^∗^ (other family members like mothers). Abbreviations: COR: crude odds ratio; CI: confidence interval.

**Table 3:** Bivariate analysis of socioeconomic and health service-related factors of cases and controls in Abobo Woreda, Gambella region, Southwest Ethiopia, 2019.

Variables	Category	Cases (88) No. %	Controls (176) No. %	COR (95% CI)	*p* value
Media exposure	No	54 (61.4)	86 (48.9)	1.66 (0.99-2.8)	0.056^∗^
	Yes	34 (38.6)	90 (51.1)	1	

Wealth index	Poor	41 (46.6)	46 (26.1)	3.12 (1.63-5.98)	<0.001^∗^
	Medium	27 (30.7)	60 (34.1)	1.58 (0.80-3.09)	0.186^∗^
	Rich	20 (22.7)	70 (39.8)	1	

Type of HF	Only HC	24 (27.6)	46 (26.6)	1	
	Both type	10 (11.5)	19 (11.0)	1.009 (0.4-2.51)	0.985
	Only HP	53 (60.9)	108 (62.4)	0.94 (0.52-1.70)	0.840

Accessibility of HF	≤5 km	78 (89.7)	169 (97.7)	1	
	>5 km	9 (10.3)	4 (2.3)	4.88 (1.46-16.32)	0.010^∗^

Means of transportation	No	34 (38.6)	48 (27.3)	1.68 (0.98-2.89)	0.061^∗^
	Yes	54 (61.4)	128 (72.7)	1	

Note: ^∗^significant at *p* < 0.25. Abbreviations: km: kilometer; COR: crude odds ratio; CI: confidence interval.

**Table 4:** Bivariate analysis of need-based factors of cases and controls in Abobo Woreda, Gambella region, Southwest Ethiopia, 2019.

Variable	Category	Cases (88) No. %	Controls (176) No. %	COR (95% CI)	*p* value
ANC visit	No	48 (54.5)	34 (19.3)	5.01 (2.86-8.79)	<0.001^∗^
	Yes	40 (45.5)	142 (80.7)	1	

History of obstetric complication	No	80 (90.9)	156 (88.6)	1.28 (0.54-3.04)	0.573
	Yes	8 (9.1)	20 (11.4)	1	

Plan for last pregnancy	No	44 (50)	76 (43.2)	1.32 (0.79-2.19)	0.295
	Yes	44 (50)	100 (56.8)	1	

Note: ^∗^significant at *p* value <0.25. Abbreviations: COR: crude odds ratio; CI: confidence interval.

**Table 5:** Multivariable analysis for determinant factors of home delivery among mothers in Abobo District, Gambella region, Southwest Ethiopia, 2019.

Variable	Category	Cases No. %	Controls No. %	COR (95% CI)	AOR (95% CI)	*p* value
Mothers educational status	No formal education	42 (47.7)	29 (16.5)	4.94 (2.53-9.66)	5.07 (2.18-11.50)	<0.001^∗^
	Primary (1–8)	24 (27.3)	72 (40.9)	1.14 (0.59-2.20)	1.39 (0.65-3.02)	0.394
	Secondary and above	22 (25.0)	75 (42.6)	1	1	

Knowledge on obstetric complications	Poor	62 (70.5)	58 (33.0)	4.85 (2.78-8.46)	3.83 (1.98-7.40)	<0.001 ^∗^
	Good	26 (29.5)	118 (67.0)	1	1	

Attitude towards delivery services	Negative	58 (65.9)	51 (29.0)	4.74 (2.74-8.19)	3.25 (1.70-6.19)	<0.001^∗^
	Positive	30 (34.1)	125 (71.0)	1	1	

Wealth index	Poor	41 (46.6)	46 (26.1)	3.12 (1.63-5.98)	4.55 (2.01-10.31)	<0.001^∗^
	Medium	27 (30.7)	60 (34.1)	1.58 (0.80-3.09)	1.78 (0.78-4.07)	0.172
	Rich	20 (22.7)	70 (39.8)	1	1	

ANC visit	No	48 (54.5)	34 (19.3)	5.01 (2.86-8.79)	3.29 (1.63-6.63)	0.001^∗^
	Yes	40 (45.5)	142 (80.7)	1	1	

Note: ^∗^statically significant at *p* value <0.05. Abbreviations: AOR: adjusted odds ratio; COR: crude odds ratio; CI: confidence interval.

## Data Availability

The datasets generated and/or analyzed during the current study are available from the corresponding author on the reasonable request.

## Abbreviations

ANC: Antenatal care
AOR: Adjusted odds ratio
CI: Confidence interval
COR: Crude odds ratio
EDHS: Ethiopian Demographic Health Survey
HC: Health center
HF: Health facility
HP: Health post
HSTP: Health sector transformation plan
km: Kilometer
MM: Maternal mortality
MMR: Maternal mortality ratio
PCA: Principal component analysis
SDG: Sustainable Development Goal
SPSS: Statistical Package for Social Science
VIF: Variance inflation factor.
